# Assessment of potential risk factors for new onset disabling low back pain in Japanese workers: findings from the CUPID (cultural and psychosocial influences on disability) study

**DOI:** 10.1186/s12891-017-1686-y

**Published:** 2017-08-02

**Authors:** Mika Kawaguchi, Ko Matsudaira, Takayuki Sawada, Tadashi Koga, Akiko Ishizuka, Tatsuya Isomura, David Coggon

**Affiliations:** 1 Clinical Study Support, Inc., 2F Daiei Bldg., 1-11-20 Nishiki, Naka-ku, Nagoya, 460-0003 Japan; 2 Department of Medical Research and Management for Musculoskeletal Pain, 22nd Century Medical and Research Center, Faculty of Medicine, The University of Tokyo, Tokyo, Japan; 3 Shin Nippon Biomedical Laboratories, Ltd., Kagoshima, Japan; 4 Institute of Medical Science, Tokyo Medical University, Tokyo, Japan; 5 MRC Lifecourse Epidemiology Unit, Southampton General Hospital, University of Southampton, Southampton, UK; 6 Arthritis Research UK/MRC Centre for Musculoskeletal Health and Work, University of Southampton, Southampton, UK

**Keywords:** New onset, Disabling low back pain, Prospective study, Risk factors, Japanese workers, Symptom-free

## Abstract

**Background:**

Most studies of risk factors for new low back pain (LBP) have been conducted in Western populations, but because of cultural and environmental differences, the impact of causal factors may not be the same in other countries. We used longitudinal data from the Cultural and Psychosocial Influences on Disability (CUPID) study to assess risk factors for new onset of disabling LBP among Japanese workers.

**Methods:**

Data came from a 1-year prospective follow-up of nurses, office workers, sales/marketing personnel, and transportation workers, initially aged 20–59 years, who were employed in or near Tokyo. A baseline questionnaire included items on past history of LBP, personal characteristics, ergonomic work demands, and work-related psychosocial factors. Further information about LBP was collected at follow-up. Analysis was restricted to participants who had been free from LBP during the 12 months before baseline. Logistic regression was used to assess baseline risk factors for new onset of disabling LBP (i.e. LBP that had interfered with work) during the 12 months of follow-up.

**Results:**

Among 955 participants free from LBP during the 12 months before baseline, 58 (6.1%) reported a new episode of disabling LBP during the 12-month follow-up period. After mutual adjustment in a multivariate logistic regression analysis, which included the four factors that showed associations individually (*p* < 0.1) in analyses adjusted only for gender and age, the highest odds ratio (OR) was for past history of LBP (2.8, 95% [confidence interval {CI}]: 1.6–4.9), followed by working ≥60 h per week (1.8, 95% CI: 1.0–3.5) and lifting weights ≥25 kg by hand (1.6, 95% CI: 0.9–3.0). When past history of LBP was excluded from the model, ORs for the remaining risk factors were virtually unchanged.

**Conclusions:**

Our findings suggest that among Japanese workers, as elsewhere, past history of LBP is a major risk factor for the development of new episodes of disabling back pain. They give limited support to the association with occupational lifting that has been observed in earlier research, both in Japan and in Western countries. In addition, they suggest a possible role of long working hours, which merits further investigation.

## Background

Low back pain (LBP) affects most adults at some point in their lives, some 85–95% of cases being classed as ‘non-specific’ (i.e. without identifiable underlying pathology) [1, 2]. In recent decades, it has consistently been the leading cause globally of years lived with disability [3], and in Japan, it is one of the most common causes of disability, with a reported lifetime prevalence of more than 80% [4]. In the workplace, it is a costly problem, not only impairing the health of employees, but also reducing productivity [5]. The largest societal costs arise from cases in which the pain is disabling [6].

Various risk factors for the development of LBP have been identified previously, including mechanical stress from occupational activities such as lifting, bending, twisting and manual handling [7], and also psychosocial factors such as low mood, somatizing tendency (a tendency to worry about common somatic symptoms), job dissatisfaction, and adverse health beliefs about the causes and prognosis of back disorders [7–12]. Moreover, epidemiological studies indicate that most people with a history of LBP experience a recurrence within a year [13–16]. Thus, the occurrence of LBP is an important predictor of future episodes [7, 8, 17–20].

Most of the research on these risk factors has been conducted in Western populations, but it is possible that because of cultural and environmental differences, their impacts are not the same in other countries [21]. In an earlier prospective cohort study of Japanese workers who had been symptom-free for at least 1 year, we found that, in accordance with observations in Western populations [7, 22–24], past history of LBP, interpersonal stress at work, and frequent occupational lifting were all important predictors of disabling LBP [25]. Before that study, risk factors for new onset LBP, and in particular the role of psychosocial aspects of work, had not been properly assessed through prospective epidemiological research in Japan, and there remains a need for further investigation to confirm its findings.

We therefore conducted a new longitudinal study, as part of an international investigation of risk factors for musculoskeletal pain and associated disability, the Cultural and Psychosocial Influences on Disability (CUPID) study, which focused on workers aged 20–59 years from 47 occupational groups in 18 countries [26–30]. Using data from the CUPID study, we again assessed risk factors for new onset of disabling LBP among Japanese workers.

## Methods

### Study design

Our target population for the present study was Japanese workers. We used data from a 1-year prospective follow-up of Japanese participants in the CUPID study, which were collected from four groups of workers employed in or near Tokyo: nurses from a university hospital; office workers in administrative and clerical jobs at the same hospital, four pharmaceutical companies and a privately-owned trading company; sales/marketing personnel from six pharmaceutical companies; and transportation workers (mainly lorry drivers and loaders) from two courier companies transporting baggage and mail.

### Data collection

At each participating organization, a self-administered questionnaire with a covering letter from the study team was distributed to all employees in specified jobs. Workers were asked to return the completed questionnaire by post directly to the study administration office, including their name and mailing address for the purpose of follow-up. During 2009, a total of 3187 baseline questionnaires were distributed (nurses: 1074; office workers: 425; sales/marketing personnel: 380; transportation operatives: 1308), and of these, 2651 (83.2%) were completed and returned. After approximately 1 year, a follow-up questionnaire was sent to those participants who had returned the baseline questionnaire and consented to further contact. Of the 2651 participants who completed the baseline questionnaire, 1809 (68.2%) returned satisfactory follow-up questionnaires.

### Baseline questionnaire

The baseline questionnaire comprised a Japanese translation of the original CUPID questionnaire [26], supplemented with additional questions for Japanese workers. Accuracy of translation was checked by independent back-translation into English.

Among other things, the questionnaire assessed the occurrence of LBP during the past 12 months, experience of LBP more than 12 months earlier (past history of LBP), and various individual and work-related risk factors [6]. LBP was defined as occurring in an area between the costal margin and inferior gluteal folds that was depicted in a diagram [26]. Severity of LBP was classified to four grades, based on a scheme devised by Von Korff: grade 0 (no LBP), grade 1 (LBP not interfering with work), grade 2 (LBP interfering with work), and grade 3 (LBP interfering with work and leading to sick-leave) [31].

The baseline questionnaire also assessed various personal characteristics (age, gender, age at which full-time education was finished, marital status, obesity [body mass index {BMI} ≥ 25 kg/m^2^], smoking habits, habitual exercise), tenure of current job, hours worked per week, whether an average working day entailed lifting weights of ≥25 kg by hand, work-related psychosocial factors (interpersonal stress at work, inadequate breaks, job control, support from others when at work, job satisfaction), mental health, emotional trauma in childhood, awareness of colleagues and family members with LBP, somatizing tendency, and adverse beliefs about LBP.

Smoking was quantified in terms of the Brinkman Index (calculated as the product of the total number of cigarettes smoked per day and the duration of smoking in years) [32]. Individuals with a Brinkman Index of ≥400 were classed as heavy smokers, and the remainder (including non-smokers) as non-heavy smokers.

Work-related psychosocial factors were each assessed through a single question. Questions on interpersonal stress and inadequate breaks were supplementary to the original English version of the CUPID questionnaire, and allowed for two possible answers – yes or no. Job control was defined as lacking when participants reported “seldom” or “never/almost never” having choice in deciding how to work. Support at work was classed as lacking in those who said that they “seldom” or “never” received help or support from colleagues when they encountered difficulties in their work. Job dissatisfaction was deemed to occur when in response a question about the extent to which they had been satisfied with their job as whole taking everything into consideration, participants answered “dissatisfied” or “very dissatisfied”.

To assess mental health, relevant items from the MOS 36-item short-form health survey (SF-36) ver.1.2 were used [33, 34]. A score of 52 or lower on the SF-36 ver.1.2 mental health summary was taken to indicate depressed mood, 52 being the cut-point for diagnosing depression in Japanese adults [35].

Somatizing tendency was assessed using questions from the Brief Symptom Inventory [36], and was graded according to the number of symptoms (0, 1, ≥2) from a total of five (faintness or dizziness, pains in the heart or chest, nausea or upset stomach, trouble getting breath, hot or cold spells) that were reported as at least moderately distressing in the past week.

Adverse beliefs about LBP were assessed through questions derived from the Fear Avoidance Beliefs Questionnaire [37]. Participants were classed as having adverse beliefs about physical activity if they completely agreed that for someone with LBP, physical activity should be avoided as it might cause harm and that rest is needed to get better. They were deemed to have adverse beliefs about work-relatedness if they completely agreed that LBP is commonly caused by work. And they were considered to have adverse beliefs about prognosis if they completely agreed that neglecting LBP can cause permanent health problems and completely disagreed that such problems usually get better within 3 months.

### Follow-up questionnaire

The follow-up questionnaire included items on any change of job since baseline, and the presence and severity of LBP in the past 12 months. The severity of LBP was graded in the same way as at baseline.

### Eligibility criteria

In our analysis for this report, we restricted our attention to participants who had been free from LBP for the past 12 months at baseline, and who did not change their job during the follow-up period.

### Outcome

The outcome of interest was any new onset of disabling LBP during the 12 months of follow-up, where pain was defined as disabling if it had interfered with work (grade 2 or 3).

### Statistical methods

Descriptive statistics were calculated, and then logistic regression was used to explore associations with risk factors. These were summarised by odds ratios (ORs) with 95% confidence intervals (CIs). First, each risk factor was analysed separately: a) with adjustment only for age and gender; and b) with adjustment also for past history of LBP, which had been identified as an important risk factor in earlier research including our own [7, 25]. Risk factors with *p*-values <0.1 when adjusted only for age and gender were then taken forward for inclusion in a single multivariate model with mutual adjustment. The software package SAS Release 9.3 (SAS Institute, Cary, NC) was used for all statistical analyses.

### Ethical approval

Ethical approval for the study was obtained from the ethics committees of the University of Tokyo Hospital and review board of the Japan Labour Health and Welfare Organization. All participants provided written informed consent.

## Results

### Baseline characteristics of the study participants

**Fig. 1:**
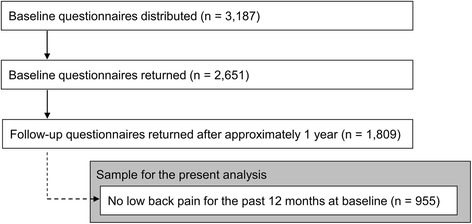
Flow chart of the sample selection for the present analysis

Of the 1809 participants who responded to the 1-year follow-up questionnaire, 955 had reported no LBP during the previous 12 months at baseline, and were included in subsequent analyses (Fig. 1). Their mean (standard deviation: SD) age at baseline was 36.7 (9.9) years, most were male (*n* = 651; 68.3%), and they had a mean (SD) BMI at baseline of 22.2 (3.0) kg/m^2^. The proportions by occupational group were: transportation operatives (38.1%), nurses (23.8%), sales/marketing personnel (21.1%), and office workers (16.7%).

### Incidence of new onset disabling low back pain

Among the 955 eligible participants, 58 (6.1%) reported a new episode of disabling LBP during the 12-month follow-up period. Their mean (SD) age at baseline was 34.4 (8.7) years, and most were male (62.1%). In most cases the severity was graded 2 (*n* = 43, 74.1%), but 15 (25.9%) had grade 3 LBP. Among the latter, total sick-leave during the 12 months was mostly 1–5 days (73.3%), while the rest had been absent for 6–30 days (26.7%).

### Association of new onset disabling low back pain with risk factors

Table 1 shows ORs for the onset of disabling LBP, after adjustment for age and gender, and then also for past history of LBP. In the analyses adjusted only for gender and age, four factors were associated with *p*-values <0.1, and thus met the criterion for inclusion in subsequent multivariate analysis. These were: past history of LBP (OR: 2.6, 95% CI: 1.5–4.6), working ≥60 h per week (OR: 2.1, 95% CI: 1.1–4.0), lifting weights of ≥25 kg by hand (OR: 1.9, 95% CI: 1.1–3.3), and inadequate breaks (OR: 1.8, 95% CI: 1.0–3.1). When associations were adjusted also for past history of LBP, working ≥60 h per week (OR: 2.0, 95% CI: 1.1–3.9) and lifting weights ≥25 kg by hand (OR: 1.9, 95% CI: 1.1–3.3) remained the strongest risk factors.

**Table 1:** Associations of risk factors at baseline with new onset of disabling low back pain

Risk factor	^a^n (%)	^b^OR	(95% CI)	^c^OR	(95% CI)
Age					
< 40 years	618 (65.2)				
40–49 years	200 (21.1)				
≥ 50 years	130 (13.7)				
Female gender	302 (31.7)				
Past history of LBP	313 (33.8)	2.6	(1.5–4.6)*		
Finished full-time education before age 19 years	304 (31.9)	1.4	(0.8–2.6)	1.4	(0.7–2.7)
BMI ≥ 25 kg/m^2^ (obesity)	133 (14.2)	1.1	(0.5–2.5)	0.9	(0.4–2.1)
< 5 h sleep per day	82 (8.7)	1.8	(0.8–3.9)	1.5	(0.6–3.4)
Not married	445 (46.9)	0.8	(0.4–1.4)	0.8	(0.4–1.4)
Heavy smoker	133 (13.9)	0.8	(0.4–1.5)	0.6	(0.2–1.9)
Employed in current job for <1 year	96 (10.1)	1.2	(0.5–2.7)	1.3	(0.6–3.1)
Work ≥60 h per week	364 (38.8)	2.1	(1.1–4.0)*	2.0	(1.1–3.9)*
Lift weights ≥25 kg by hand	452 (47.3)	1.9	(1.1–3.3)*	1.9	(1.1–3.3)*
Aware of colleague(s) with LBP	687 (72.5)	1.2	(0.6–2.2)	1.1	(0.6–2.2)
Aware of family member(s) with LBP	301 (31.5)	1.2	(0.7–2.2)	1.1	(0.6–2.0)
Irregular work shifts	304 (31.9)	1.1	(0.6–2.0)	1.1	(0.6–1.9)
Interpersonal stress at work	458 (48.0)	1.3	(0.7–2.2)	1.1	(0.6–1.9)
Inadequate breaks at work	507 (53.1)	1.8	(1.0–3.1)	1.6	(0.9–2.9)
Lack of job control	347 (36.4)	0.9	(0.5–1.5)	0.8	(0.5–1.5)
Lack of support at work	72 (7.7)	2.0	(0.8–4.6)	1.9	(0.8–4.6)
Dissatisfied with job	378 (39.7)	0.8	(0.4–1.4)	0.7	(0.4–1.3)
Low mood	265 (28.0)	1.0	(0.6–1.9)	1.0	(0.5–1.8)
Regular exercise < once per week	652 (69.3)	1.0	(0.6–1.8)	0.9	(0.5–1.7)
Emotional trauma in childhood	66 (7.1)	2.0	(0.9–4.7)	1.7	(0.7–3.9)
Number distressing somatic symptoms					
0	760 (80.3)	1.0		1.0	
1	132 (13.9)	1.3	(0.6–2.6)	1.4	(0.7–2.9)
≥ 2	55 (5.8)	0.3	(0.0–1.9)	0.3	(0.0–2.0)
Adverse beliefs about LBP					
Work relatedness	306 (32.3)	1.3	(0.8–2.3)	1.3	(0.8–2.3)
Physical activity	208 (22.0)	1.0	(0.5–1.9)	1.1	(0.5–2.0)
Prognosis	155 (16.4)	0.8	(0.4–1.7)	0.9	(0.4–1.9)

Totals may not sum to 100% due to rounding

*OR* odds ratio; *CI* confidence interval; *LBP* low back pain

^a^Data on individual risk factors were missing for up to 29 participants. Each logistic regression analysis was limited to participants with complete information on all of the risk factors included in the relevant model

^b^Odds ratios (with 95% confidence intervals) adjusted for age and gender

^c^Odds ratios (with 95% confidence intervals) adjusted for age, gender and past history of LBP

*P < 0.05. A cut-point of *P* < 0.1 was adopted to select risk factors for inclusion in a subsequent multivariate model (see Table 2)

After mutual adjustment in multivariate logistic regression analysis, the ORs were a little lower overall, but with a similar pattern to that in the earlier analyses (Table 2). The highest OR was for past history of LBP (OR: 2.8, 95% CI: 1.6–4.9), followed by working ≥60 h per week (OR: 1.8, 95% CI: 1.0–3.5) and lifting weights ≥25 kg by hand (OR: 1.6, 95% CI: 0.9–3.0). When past history of LBP was excluded from the model, ORs were virtually unchanged.

**Table 2:** Mutually adjusted associations of risk factors at baseline with new onset of disabling low back pain

Risk factor	^a^OR	(95% CI)	^b^OR	(95% CI)
Age				
< 40 years	1.0		1.0	
40–49 years	0.8	0.4–1.8	1.0	0.5–2.0
≥ 50 years	0.7	0.2–1.9	0.7	0.2–2.1
Female gender	1.4	0.7–2.8	1.5	0.8–3.0
Work ≥60 h per week	1.8	1.0–3.5	1.9	1.0–3.6
Lift weights ≥25 kg by hand	1.6	0.9–3.0	1.5	0.8–2.8
Inadequate break time at work	1.4	0.7–2.6	1.4	0.8–2.7
Past history of LBP	2.8	1.6–4.9	–	–

Participants with missing data for any of the variables in the model were excluded

^a^Mutually adjusted odds ratios (with 95% confidence intervals) derived from a logistic regression model which included all of the variables for which results are presented

^b^Mutually adjusted odds ratios (with 95% confidence intervals) derived from a logistic regression model which included all of the variables for which results are presented but did not adjust for past history of LBP

## Discussion

These results indicate that past history of LBP and working long hours were risk factors for the new onset of disabling LBP among Japanese workers who had been symptom-free during the 12 months before baseline. In addition, risk was increased in participants who reported occupational lifting, although not significantly at a 5% level.

In the present investigation, the incidence of disabling LBP was relatively low (6.1%) which may reflect our strict definition of disability (interference with work), as well as the requirement for a long symptom-free period before baseline. It has previously been proposed that an episode of LBP can be classed as new if it occurs after a period of at least 1–3 months without symptoms [38]. However, LBP is commonly recurrent within a year [13–16]. Moreover, a recent systematic review indicated that only 33% of patients in a primary care setting have recovered from non-specific LBP at a year after onset, whereas approximately 65% still experience pain [39]. Give these findings, we felt justified in requiring a 12-month symptom-free period at baseline, when exploring risk factors for new episodes, although we recognize that the criteria are to some extent arbitrary. In our earlier study, the incidence of new disabling LBP during 2 years of follow-up in workers who had been without LBP for more than 12 months before baseline was 3.9%, which is a little lower than in the current investigation [25].

We found that past history of LBP was the strongest and most significant risk factor for new disabling LBP, with an OR of almost three. This accords with our earlier study in Japan [25], and also with observations in Western populations [7, 8, 17–20]. It may be that the occurrence of a back problem renders an individual more vulnerable to future episodes of LBP (e.g. through changes in spinal structure and function or in the central processing of pain). Alternatively, the association might reflect continuing exposure to risk factors that were responsible for the initial development of the back problem. In our analysis, the association with past history of LBP was present after adjustment for other risk factors, but there may have been other important determinants of LBP that we did not assess.

In addition to past history of LBP, working ≥60 h per week and lifting weights of ≥25 kg by hand carried significantly elevated risk in analyses that adjusted for age and gender, the association with occupational lifting falling just short of significance when risk estimates were mutually adjusted. Biomechanical loading of the spine from manual handling tasks such as lifting, has been found experimentally to be greater in the presence of demands for mental processing that induce stress [24, 40]. Moreover, working overtime has been reported to increase risk of musculoskeletal disorders such as LBP [41]. While excessive working hours, perhaps entailing physical exhaustion as well as mental strain, could of itself lead to LBP, it might also act by potentiating the risks from spinal strain as a consequence of heavy lifting.

Long working hours may also reflect an element of “workaholism” in which an employee, whether for personal reasons or in response to an over-demanding job, spends excessive time at work to the detriment of his or her personal life [42]. This too is a previously reported risk factor for disabling LBP [43].

An association with long working hours was not apparent in our earlier study [25]. On the other hand, that investigation found new incidence of disabling LBP aside was significantly related to interpersonal stress at work, a finding that was not replicated in the current analysis. These differences may reflect differing characteristics of the populations studied. For example, in the earlier investigation, the participants were mostly male (88.3%) and office workers (76.1%). Alternatively, they could have occurred by chance. They underline the need for replication of results, especially when multiple risk factors are examined without strong prior expectations, and there is therefore greater potential for false positive results.

That said, the findings of the present study are not clearly different from those in Western populations. Divergence from other countries in the factors affecting new onset of disabling LBP might perhaps have been expected as a consequence of cultural differences. However, a trend to westernization in Japan may have reduced those differences. Alternatively, our questionnaire may not have covered risk factors that would differ from those in other countries or cultures.

Some limitations of our investigation should be noted. First, the generalizability of the results may be limited because the study sample was not fully representative. For example, the proportion of female participants was small in comparison with that in the national workforce of Japan. Second, because information about exposures and symptoms was obtained by self-report, some degree of misclassification is likely. Physical exposures, such as heavy lifting, might be assessed better using objective measures. Because of constraints on the total length of the questionnaire, the ascertainment of interpersonal stress was based on a single question rather than the longer Brief Job Stress Questionnaire [44] that we had used to assess psychosocial factors including interpersonal stress in our earlier study. In addition, there is a possibility of recall bias, given that the presence and severity of LBP, both at baseline and follow-up, were ascertained retrospectively. For example, participants with physically demanding jobs may have been more likely to recall symptoms and difficulty with work. Third, because the outcome was relatively infrequent, statistical power was limited. Lastly, although the present analysis included most of the well-established risk factors for new onset LBP, as well as other potential risk factors that have been suggested by earlier studies, it is possible that some important determinants, perhaps distributed differentially by occupational group, were overlooked, leading to unrecognized residual confounding. Given these limitations, our results should be interpreted with caution.

## Conclusion

In conclusion, our findings suggest that among Japanese workers, as elsewhere, past history of LBP is a major risk factor for the development of new episodes of disabling back pain. They give limited support to the association with occupational lifting that has been observed in earlier research, both in Japan and in Western countries. In addition, they suggest a possible role of long working hours, which merits further investigation.

## Abbreviations

BMI: Body mass index
CI: Confidence interval
CUPID: Cultural and Psychosocial Influences on Disability
LBP: Low back pain
OR: Odds ratio
SD: Standard deviation
SF-36: MOS 36-item short-form health survey

## References

[1] Krismer M, van Tulder M (2007). Low back pain (non-specific). Best Pract Res Clin Rheumatol.

[2] Deyo RA, Rainville J, Kent DL (1992). What can the history and physical examination tell us about low back pain?. JAMA.

[3] Vos T, Flaxman AD, Naghavi M, Lozano R, Michaud C, Ezzati M (2012). Years lived with disability (YLDs) for 1160 sequelae of 289 diseases and injuries 1990-2010: a systematic analysis for the global burden of disease study 2010. Lancet.

[4] Fujii T, Matsudaira K (2013). Prevalence of low back pain and factors associated with chronic disabling back pain in Japan. Eur Spine J.

[5] Feldman JB (2004). The prevention of occupational low back pain disability: evidence-based reviews point in a new direction. J Surg Orthop Adv.

[6] Snook SH (2004). Work-related low back pain: secondary intervention. J Electromyogr Kinesiol.

[7] Waddell G, Burton AK (2001). Occupational health guidelines for the management of low back pain at work: evidence review. Occup Med.

[8] Papageorgiou AC, Croft PR, Thomas E, Ferry S, Jayson MI, Silman AJ (1996). Influence of previous pain experience on the episode incident of low back pain: results the South Manchester back pain study. Pain.

[9] Pincus T, Burton AK, Vogel S, Field AP (2002). A systematic review of psychological factors as predictors of chronicity/disability in prospective cohorts of low back pain. Spine (Phila Pa 1976).

[10] Hoogendoorn WE, van Poppel MN, Bongers PM, Koes BW, Bouter LM (2000). Systematic review of psychosocial factors at work and private life as risk factors for back pain. Spine (Phila Pa 1976).

[11] Linton SJ (2001). Occupational psychological factors increase the risk for back pain: a systematic review. J Occup Rehabil.

[12] Farioli A, Mattioli S, Quaglieri A, Curti S, Violante FS, Coggon D (2014). Musculoskeletal pain in Europe: the role of personal, occupational, and social risk factors. Scand J Work Environ Health.

[13] Carey TS, Garrett JM, Jackman A, Hadler N (1999). Recurrence and care seeking after acute back pain: results of a long-term follow-up study. North Carolina back pain project. Med Care.

[14] Pengel L, Herbert R, Maher CG, Refshauge KM. Acute low back pain: a systematic review of its prognosis. BMJ. 2003;327:323–7. 10.1136/bmj.327.7410.323 PMC169642 12907487

[15] Von Korff M (1994). Studying the natural history of back pain. Spine.

[16] Von Korff M, Deyo RA, Cherkin DC, Barlow W (1993). Back pain in primary care: outcomes at 1 year. Spine.

[17] Burton AK, Balagué F, Cardon G, Eriksen HR, Henrotin Y, Lahad A, COST B13 Working Group on European Guidelines for Prevention in Low Back Pain et al. How to prevent low back pain. Best Pract Res Clin Rheumatol 2005;19:541–555. 10.1016/j.berh.2005.03.001 15949775

[18] Hestbaek L, Leboeuf-Yde C, Kyvik KO (2006). Is comorbidity in adolescence a predictor for adult low back pain? A prospective study of a young population. BMC Musculoskelet Disord.

[19] Harreby M, Kjer J, Hesselsøe G, Neergaard K (1996). Epidemiological aspects and risk factors for low back pain in 38-year-old men and woman: a 25-year prospective cohort study of 640 school children. Eur Spine J.

[20] Smedley J, Egger P, Cooper C, Coggon D (1997). Prospective cohort study of predictors of incident low back pain in nurses. BMJ.

[21] Waddell G, Waddell G (2004). Social interactions. The back pain revolution.

[22] Linton SJ, Nachemson AJ, Jonsson E (2000). Psychological risk factors for neck and back pain. Neck and back pain: the scientific evidence of causes, diagnosis and treatment.

[23] Harkness EF, Macfarlane GJ, Nahit ES, Silman AJ, McBeth J (2003). Risk factors for new-onset low back pain amongst cohorts of newly employed workers. Rheumatology.

[24] Davis KG, Marras WS, Heaney CA, Waters TR, Gupta P (2002). The impact of mental processing and pacing on spine loading. Spine.

[25] Matsudaira K, Konishi H, Miyoshi K, Isomura T, Takeshita K, Hara N (2012). Potential risk factors for new onset of back pain disability in Japanese workers: findings from the Japan epidemiological research of occupation-related back pain study. Spine.

[26] Coggon D, Ntani G, Palmer KT, Felli VE, Harari R, Barrero LH (2012). The CUPID (cultural and psychosocial influences on disability) study: methods of data collection and characteristics of study sample. PLoS One.

[27] Coggon D, Ntani G, Vargas-Prada S, Martinez JM, Serra C, Benavides FG, Members of CUPID Collaboration (2013). International variation in absence from work attributed to musculoskeletal illness: findings from the CUPID study. Occup Environ Med.

[28] Matsudaira K, Palmer KT, Reading I, Hirai M, Yoshimura N, Coggon D (2011). Prevalence and correlates of regional pain and associated disability in Japanese workers. Occup Environ Med.

[29] Coggon D, Ntani G, Palmer KT, Felli VE, Harari R, Barrero LH (2013). Patterns of multisite pain and associations with risk factors. Pain.

[30] Fujii T, Matsudaira K, Yoshimura N, Hirai M, Tanaka S (2013). Associations between neck and shoulder discomfort (Katakori) and job demand, job control, and worksite support. Mod Rheumatol.

[31] Von Korff M, Ormel J, Keefe FJ, Dworkin SF (1992). Grading the severity of chronic pain. Pain.

[32] Brinkman GL, Coates O (1963). The effect of bronchitis, smoking and occupation on ventilation. Ann Rev Respir Dis.

[33] Fukuhara S, Bito S, Green J, Hsiao A, Kurokawa K (1998). Translation, adaptation, and validation of the SF-36 health survey for use in Japan. J Clin Epidemiol.

[34] Fukuhara S, Ware JE, Kosinski M, Wada S, Gandek B (1998). Psychometric and clinical tests of validity of the Japanese SF-36 health survey. J Clin Epidemiol.

[35] Yamazaki S, Fukuhara S, Green J (2005). Usefulness of five-item and three-item mental health inventories to screen for depressive symptoms in the general population of Japan. Health Qual Life Outcomes.

[36] Waddell G, Newton M, Henderson I, Somerville D, Main CJ (1993). A fear-avoidance beliefs questionnaire (FABQ) and the role of fear-avoidance beliefs in chronic low back pain and disability. Pain.

[37] Derogatis LR, Melisaratos N (1983). The brief symptom inventory: an introductory report. Psychol Med.

[38] de Vet HCW, Heymans MW, Dunn KM, Pope DP, van der Beek AJ, Macfarlane GJ (2002). Episode of low back pain. A proposal for uniform definition to be used in research. Spine.

[39] Itz CJ, Geurts JW, van Kleef M, Nelemans P (2013). Clinical course of non-specific low back pain: a systematic review of prospective cohort studies set in primary care. Eur J Pain.

[40] Katsuhira J, Matsudaira K, Iwakiri K, Kimura Y, Ohashi T, Ono R (2013). Effect of mental processing on low back load while lifting an object. Spine.

[41] Koda S, Yasuda N, Sugihara Y, Ohara H, Udo H, Otani T (2000). Analyses of work-relatedness of health problems among truck drivers by questionnaire survey. Sangyo Eiseigaku Zasshi.

[42] Scott KS, Moore KS, Miceli MP (1997). An exploration of the meaning and consequences of Workaholism. Human Relations.

[43] Matsudaira K, Shimazu A, Fujii T, Kubota K, Sawada T, Kikuchi N (2013). Workaholism as a risk factor for depressive mood, disabling back pain, and sickness absence. PLoS One.

[44] Shimomitsu T, Yokoyama K, Ono Y, Maruta T, Tanigawa T, Kato S (1998). Development of a novel brief job stress questionnaire. Report of the research grant for the prevention of work-related diseases from the Ministry of Labour.

